# FPM2Stain Net: physics-guided super-resolution and multi-modal virtual staining for digital histopathology

**DOI:** 10.1364/BOE.586327

**Published:** 2026-01-30

**Authors:** Qijun Yang, Lintao Xiang, Chang Bian, Yating Huang, Hongpei Zheng, Hujun Yin

**Affiliations:** 1 Department of Electrical and Electronic Engineering, The University of Manchester, Oxford Road, Manchester, M13 9PL, UK; 2 Division of Informatics, Imaging and Data Sciences, School of Health Sciences, The University of Manchester, Oxford Road, Manchester, M13 9PL, UK; 3 yang.qijun@hotmail.com

## Abstract

We propose FPM2Stain Net, a jointly optimized end-to-end computational pipeline that integrates physics-guided super-resolution with deep learning–based virtual staining to enable high-resolution, multi-modal digital histopathology. The first stage, bidirectional physics-based Fourier ptychographic microscopy (BiP-FPM), reconstructs high-resolution amplitude and phase maps from a multi-LED FPM image stack using a self-supervised ResNet–U-Net guided by a differentiable point spread function (PSF) model with learnable pupil correction and bidirectional physics-consistent constraints. The second stage employs a multi-task conditional generative adversarial network (cGAN) enhanced with wavelet-based spatial–frequency fusion and perceptual supervision to synthesize clinically relevant staining modalities, including H&E, DAPI, LAP2, and panCK. Extensive experiments on both simulated and real tissue datasets demonstrate that FPM2Stain Net outperforms conventional FPM, GAN-based methods, and diffusion-based models in both reconstruction fidelity and staining accuracy. The synthesized virtual stains not only preserve fine structural details but also enable downstream analysis, such as cell segmentation and biomarker quantification, with higher accuracy than using images acquired with a conventional 40× objective microscope. These results confirm that FPM2Stain Net achieves a greater-than-10× pixel-level upsampling factor relative to the low-magnification (4×) input image, and provides a fast, scalable, and cost-effective alternative to chemical staining in digital pathology, multiplex imaging, and point-of-care diagnostics.

## 1. Introduction

Fourier Ptychographic Microscopy (FPM) has emerged as a computational imaging technique that enables high-resolution, wide field-of-view imaging by synthetically expanding the spatial frequency spectrum from low-resolution images captured under coded illumination [1,2]. With its ability to recover quantitative phase and amplitude information from intensity-only measurements, FPM offers a powerful, label-free imaging solution for biomedical research and digital pathology [3,4].

**Fig. 1.**
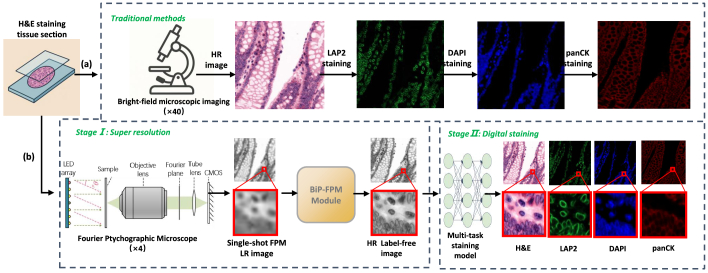
Comparison between traditional staining workflow (a) and FPM2Stain Net pipeline (b).

However, phase-based or structural outputs from FPM are often difficult to interpret in clinical settings, where diagnostic decisions rely heavily on chemically stained histological or immunofluorescence images [4,5]. Bridging this interpretability gap is critical for the clinical translation of FPM. Traditional workflows (e.g. Fig. 1(a)) require high-magnification microscopy and multiple rounds of immunostaining (e.g., LAP2, DAPI, panCK), resulting in high cost, prolonged processing time, and potential sample degradation.

**Fig. 2.**
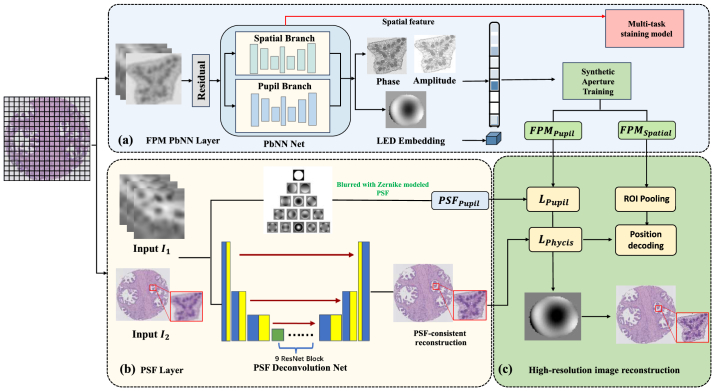
Overview of the proposed BiP-FPM pipeline. (a) The FPM PbNN Layer consists of dual spatial and pupil branches that jointly learn object amplitude, phase, and pupil function $P(f_{x},f_{y})$ from a stack of multi-LED FPM intensity images $\left\{I_{n}\right\}$ . The spatial branch encodes cross-illumination structural features, while the pupil branch estimates system aberrations via Zernike modeling. LED embedding provides illumination priors for synthetic-aperture training. (b) The PSF Layer implements a differentiable physical model that enforces bidirectional physics consistency: the forward path simulates PSF-based blurring with the learned pupil function, and the inverse path performs PSF deconvolution using a 9-block ResNet network. The two "recorrupted" outputs are compared to the real inputs to ensure forward–inverse self-supervision. (c) Estimated amplitude–phase features and corrected pupil parameters are fused for joint loss computation $\left(L_{\mathrm{phys}},L_{\mathrm{pupil}}\right)$ and subsequently fed into the multi-task staining model for high-fidelity, structurally consistent virtual staining.

To overcome these challenges, we propose a two-stage hybrid computational pipeline (Fig. 1(b)) that integrates physics-based FPM super-resolution with deep learning-driven virtual staining. The first stage leverages a novel BiP-FPM module (Fig. 2) that reconstructs high-resolution Hematoxylin & Eosin (H&E)-style structural maps from a multi-LED FPM acquisition, using a physics-informed U-Net with a learnable Zernike-modeled pupil function and a differentiable point-spread-function (PSF) layer. Bidirectional physics consistency is enforced via forward modeling and inverse deconvolution using the same optical parameters. The second stage introduces a multi-task digital staining module based on a wavelet-enhanced conditional GAN, which translates the BiP-FPM-derived structural outputs into clinically meaningful multiplex immunofluorescence (mpIF) channels—namely LAP2 (nuclear envelope), panCK (epithelial marker), and DAPI (nuclear counterstain).

Together, this end-to-end pipeline eliminates the need for multi-frame acquisition, iterative reconstruction, and/or chemical staining, providing a fast, interpretable, and cost-effective solution for label-free virtual histopathology.

The key contributions are summarized as:

• We propose BiP-FPM, a physics-informed multi-LED FPM super-resolution framework trained with bidirectional physics-consistent self-supervision (forward PSF projection and inverse PSF deconvolution) and learnable Zernike pupil correction.
• We introduce a multi-task digital staining cGAN equipped with wavelet-based spatial–frequency fusion and perceptual image quality supervision.
• We demonstrate that this integrated pipeline outperforms traditional FPM and diffusion-based staining approaches in image fidelity, biological interpretability, and clinical scalability.

## 2. Background and related work

### 2.1. Background: Fourier ptychographic microscopy

This subsection briefly summarizes the FPM forward imaging model as background for the proposed BiP-FPM, rather than reviewing classical iterative solvers in detail.

In a typical FPM setup, a thin specimen with transmission function o(r) is illuminated by an oblique plane wave generated by the *n*-th LED. The corresponding illumination wave vector is

(1) $$k_{n}=2\pi \left(\frac{\sin\theta_{xn}}{\lambda },\frac{\sin\theta_{yn}}{\lambda }\right),$$

where $\left(\theta_{xn},\theta_{yn}\right)$ denotes the illumination angle and *λ* is the wavelength. Under the *n*-th LED, the specimen spectrum is shifted as

(2) $$F\left\{o(r)\exp\left(ik_{n}\cdot r\right)\right\}=O\left(k-k_{n}\right).$$

The objective lens acts as a band-limiting pupil function P(k) , and the measured intensity is given by

(3) $$I_{n}(r)={\left|F^{-1}\left\{P(k)O\left(k-k_{n}\right)\right\}\right|}^{2}.$$

Classical FPM reconstructs a high-resolution complex field (amplitude and phase) by enforcing consistency between the forward model and the captured multi-LED measurements. In this work, we reformulate this process as a differentiable, physics-consistent learning objective (Section 3.1).

### 2.2. Related work

#### 2.2.1. FPM super-resolution

Fourier Ptychographic Microscopy (FPM) is a computational imaging technique that achieves high-resolution, wide-field imaging by synthesizing spatial frequency components captured under varied-angle LED illumination [1,3]. Its large spatial bandwidth product (SBP), low cost, and compatibility with standard microscopes make it attractive for applications such as digital pathology and hematology [4].

Traditional FPM reconstruction relies on iterative algorithms like Alternating Projections (AP), which require the sequential acquisition of dozens of low-resolution images under different illuminations. These methods are sensitive to system misalignments and aberrations that degrade the reconstruction quality [6,7]. Embedded Pupil Function Recovery (EPRY) has been introduced to compensate for aberrations during reconstruction [8], but it struggles when multiple system errors coexist.

To improve reconstruction robustness and efficiency, deep learning methods have been increasingly integrated into FPM. Jiang et al. [9] first modeled the forward imaging process of FPM with deep neural networks (DNNs), enabling data-driven image recovery. Later works expanded this by incorporating domain-specific corrections, including aberration modeling [10,11], Zernike phase correction [12], and LED misalignment compensation [13,14].

Beyond pure data-driven learning, Physics-Based Neural Networks (PbNNs) combine known physical priors with neural optimization. These approaches treat the object’s complex spectrum as trainable parameters, allowing unsupervised recovery via backpropagation guided by a differentiable forward model [15–17]. PbNNs have shown strong generalization, noise robustness, and adaptability to various imaging conditions.

A growing trend in FPM research is to reduce acquisition burden by transitioning from multi-frame to single-frame imaging. Approaches such as color-multiplexed or polarization-multiplexed illumination, combined with learned priors or initialization strategies, allow super-resolution reconstruction from just one or few images [5,18–23]. For example, Zheng et al. [22] introduced a polarization-multiplexed LED illumination strategy to enable isotropic lateral resolution in single-shot FPM, while Yoon et al. [24] proposed polarization-encoded illumination to achieve high-resolution reconstructions from a single capture. These hardware-driven solutions highlight the potential of single-shot imaging, but often require specialized optical setups. In contrast, our proposed BiP-FPM employs a deep learning-based approach by incorporating a differentiable PSF-constrained forward model into a deep learning framework, thereby enabling single-frame super-resolution reconstruction without requiring additional hardware modifications.

#### 2.2.2. PSF-based super-resolution

The point spread function (PSF) is fundamental to computational super-resolution (SR), providing a physical prior to reverse optical blurring [25–28]. Classical methods like the Richardson–Lucy (RL) deconvolution [29] and sparse deconvolution [30] utilize PSF to restore high-frequency information, but their reliance on handcrafted assumptions and manual parameter tuning limits robustness, particularly under low signal-to-noise ratio (SNR) conditions. These methods often degrade in performance when applied to biological imaging, where photon budgets and structural complexity violate their assumptions [31].

Recent advances have redefined the role of PSF by embedding it into deep learning frameworks [32]. Qiao et al. [33] proposed ZS-DeconvNet, a zero-shot unsupervised model that incorporates PSF-constrained forward models into a dual-stage neural network architecture. Without requiring any ground-truth images, ZS-DeconvNet achieves more than 1.5× resolution enhancement across multiple modalities (e.g., TIRF, SIM, LLSM), while exhibiting strong generalization to dynamic and light-sensitive samples. Complementary work has explored programmable PSF engineering through diffractive neural optics [34] and deep learning-based PSF invertibility assessment [35], further supporting a shift towards adaptive and physically grounded SR reconstruction pipelines.

#### 2.2.3. Digital staining in microscopy

In digital staining tasks, current deep learning methods primarily include convolutional neural networks (CNNs), generative adversarial networks (GANs), and diffusion models [4]. Within CNN-based approaches, U-Net [36] is widely used for cell structure prediction due to its encoder–decoder architecture tailored for biomedical segmentation, while ResNet [37] enhances the quality of synthesized images through deep feature extraction. For GAN-based methods, Pix2Pix was the first conditional GAN (cGAN) applied to digital staining [38], and CycleGAN [39] further enabled stain-to-stain image translation without paired datasets. StainGAN has also been adopted for stain normalization, facilitating improved domain adaptation across tissue slides. More recently, diffusion models have emerged as a promising alternative, offering more stable convergence, fewer hallucination artifacts, and superior performance in high-fidelity image generation tasks [40,41]. Notably, a recent study [42] proposed a Brownian Bridge Diffusion Model (BBDM) for pixel super-resolved virtual staining of label-free tissue, which not only achieves both virtual staining and spatial resolution enhancement simultaneously, but also outperforms state-of-the-art cGAN-based models in SSIM, PSNR, and LPIPS metrics. Generative models introduced sampling engineering techniques such as mean and skip sampling to reduce inference variance and demonstrate strong generalization to unseen tissue types (e.g., lung and heart), indicating its robustness and clinical applicability in digital pathology [43–56].

In parallel, Chae et al. [57] introduced a phase-to-color translation framework that restores faded histology slides into high-quality H&E stains using phase images as input, addressing a practical challenge in pathology workflows. While effective for single-modality recovery, such approaches remain limited to H&E prediction and do not generalize to multiplex immunofluorescence tasks.

Beyond deep learning, efforts have also been made to enable digital staining within Fourier Ptychographic Microscopy (FPM). Color-Transfer Fourier Ptychographic Microscopy (CFPM) and its improved variant, Color-Transfer Filtering FPM (CFFPM), transfer color textures from low-resolution RGB images to high-resolution grayscale FPM reconstructions to avoid the need for sequential tri-wavelength acquisition [18,58]. Although these methods significantly improve imaging throughput and are compatible with physical-model-based reconstruction, they remain limited by sensitivity to dye combinations, difficulties in multi-stain differentiation, and reliance on heuristic color matching without semantic understanding of tissue structures. Such limitations may undermine the consistency and interpretability of virtually stained images in downstream analytical tasks, including cell type classification and spatial quantification. In addition, the lack of semantic fidelity in color representation can compromise clinical reliability, especially in diagnostic settings where subtle color variations encode pathological significance. Therefore, while digital staining in FPM offers throughput advantages, enhancing its robustness and semantic accuracy remains critical for its translation to clinical grade digital pathology.

## 3. Methodology

### 3.1. BiP-FPM: bidirectional physics-based Fourier ptychographic microscopy

To achieve physically grounded and structurally consistent digital staining directly from raw FPM measurements, we design FPM2Stain Net as an end-to-end framework that couples a Bidirectional Physics-based Fourier Ptychographic Microscopy (BiP-FPM) module with a multi-task virtual-staining network. Unlike the previous single-frame design, BiP-FPM leverages a stack of low-resolution intensity images acquired under multiple LED illuminations, enabling physics-constrained super-resolution reconstruction without ground-truth supervision.

#### BiP-FPM Physics-based Reconstruction.

The BiP-FPM front end receives $I_{\mathrm{FPM}}={\left\{I_{n}\right\}}_{n=1}^{N}$ and passes them through a nine-block ResNet U-Net encoder–decoder to extract cross-illumination spatial–frequency representations. The decoder predicts four physically interpretable quantities: amplitude A(x,y) , phase ϕ(x,y) , pupil function $P(f_{x},f_{y})$ (Zernike-parameterized), and illumination gain *s*. The reconstructed complex field at the sample plane is

(4) $${\hat{U}}_{\mathrm{HR}}(x,y)=A(x,y)e^{j\phi (x,y)}.$$

#### Differentiable Forward Model.

To guarantee optical realism, a differentiable Fourier-optics model synthesizes each LED observation:

(5) $${\hat{I}}_{n}=s\;{\left|F^{-1}\;(P(f)\;{\tilde{U}}_{\mathrm{HR}}(f-k_{n}))\right|}^{2},$$

where ${\tilde{U}}_{\mathrm{HR}}=F\{Ae^{j\phi }\}$ and $k_{n}$ denotes the illumination shift vector determined by the *n*-th LED. The forward model preserves the physics of coherent imaging and is fully differentiable, allowing gradient flow from intensity loss back to amplitude, phase, and pupil parameters.

#### Synthetic Aperture Training.

In conventional FPM, the high-resolution spectrum is reconstructed by iteratively stitching the frequency patches collected under different illumination angles. In BiP-FPM, this process is reformulated as a differentiable synthetic aperture training mechanism that fuses the per-LED complex fields in the frequency domain. Given the predicted complex field ${\tilde{U}}_{\mathrm{HR}}(f)=F\{Ae^{j\phi }\}$ , each LED illumination shifts a sub-spectrum by its wave vector $k_{n}$ :

(6) $${\tilde{U}}_{n}(f)=P(f)\;{\tilde{U}}_{\mathrm{HR}}(f-k_{n}),$$

where P(f) is the learnable pupil function that acts as a low-pass filter. All shifted sub-spectra are then fused into a synthetic aperture to reconstruct the global high-resolution spectrum:

(7) $${\tilde{U}}_{\mathrm{syn}}(f)=\frac{1}{N}\sum_{n=1}^{N}W_{n}(f)\;P(f)\;{\tilde{U}}_{\mathrm{HR}}(f-k_{n}),$$

where $W_{n}(f)$ denotes an LED-dependent weighting mask that ensures smooth frequency transitions between overlapping regions. The corresponding high-resolution spatial reconstruction is obtained through the inverse Fourier transform:

(8) $${\hat{U}}_{\mathrm{HR}}(x,y)=F^{-1}\;[{\tilde{U}}_{\mathrm{syn}}(f)].$$

During training, the loss $L_{\mathrm{phys}}$ in Eq. (5) is computed between the simulated intensity ${\hat{I}}_{n}$ and the real measurement $I_{n}$ for all LEDs, allowing gradients to propagate across all illumination channels via the synthetic aperture formulation. This design enables BiP-FPM to jointly learn amplitude, phase, and pupil corrections while implicitly performing end-to-end spectral stitching without explicit iterative ptychographic reconstruction.

#### Bidirectional Physical Consistency.

A dual-path loss enforces both forward and inverse consistency:

(9) $$L_{\text{FPM}}=L_{\text{phys}}+\lambda_{d}L_{\text{deconv}},$$

where the forward consistency is

(10) $$L_{\text{phys}}=\frac{1}{N}\sum_{n=1}^{N}\;{\left\|I_{n}-{\hat{I}}_{n}\right\|}_{2}^{2},$$

and the inverse consistency employs PSF-based deconvolution:

(11) $$L_{\text{deconv}}={\left\|{\hat{U}}_{\mathrm{HR}}-U_{\mathrm{deconv}}\right\|}_{2}^{2},$$

(12) $$U_{\mathrm{deconv}}=F^{-1}\;\left(\frac{\overset{―}{H}(f)F\{I_{\mathrm{avg}}\}}{{\left|H(f)\right|}^{2}+\alpha }\right),$$

where H(f) is the optical-transfer function consistent with *P*, $I_{\mathrm{avg}}=\frac{1}{N}\sum_{n}I_{n}$ , and *α* is the Wiener stabilizer. This bidirectional formulation constrains BiP-FPM to remain physically invertible while promoting high-frequency recovery and noise robustness.

#### Pupil Correction and Zernike Modeling.

To compensate optical aberrations, the learnable pupil is expanded into 15 Zernike modes:

(13) $$P_{\text{Zernike}}(f)=\mathrm{circ}\;\left(\frac{∥f∥}{f_{c}}\right)\exp\;\left(j\sum_{i=1}^{15}a_{i}Z_{i}(\rho ,\theta )\right).$$

Two auxiliary reconstructions are produced for stable supervision:

(14) $$I_{\text{rec},n}^{A}=s\;{\left|F^{-1}\;(P_{\text{Zernike}}\;{\tilde{U}}_{\mathrm{HR}}(f-k_{n}))\right|}^{2},$$

(15) $$I_{\text{rec}}^{B}={\left|U_{\mathrm{deconv}}\right|}^{2}.$$

By minimizing discrepancies among $\left\{I_{n}\right\}$ , $\left\{I_{\text{rec},n}^{A}\right\}$ , and $I_{\text{rec}}^{B}$ , the network jointly enforces forward (pupil-aware) and inverse (PSF-based) physical consistency.

#### Regularization and Overall Objective.

Smoothness and optical plausibility are encouraged through total-variation and pupil regularization:

(16) $$L_{\text{BiP-FPM}}=L_{\text{phys}}+\lambda_{d}L_{\text{deconv}}+\lambda_{\mathrm{tv}}\;(\mathrm{TV}(A)+\mathrm{TV}(\phi ))+\lambda_{P}R(P),$$

where

(17) $$R(P)=\eta_{1}\;\sum_{i}{\left|a_{i}\right|}^{2}+\eta_{2}\;{\left\|\;P(f)-\overset{―}{P}(-f)\;\right\|}_{2}^{2}$$

penalizes non-smooth or asymmetric pupil functions.

#### Structural Feature Integration for Digital Staining.

The BiP-FPM encoder yields multi-scale structural features $\left\{F_{s}^{i}\right\}$ capturing tissue morphology. These are resized and projected via 1×1 convolutions:

(18) $${\hat{F}}_{s}^{i}={\mathrm{Conv}}_{1\times 1}(\mathrm{Resize}(F_{s}^{i})),i=1,2,3,$$

and fused with frequency-domain features $F_{f}$ within the frequency–spatial fusion (FS-FF) module:

(19) $$F_{\text{out}}={\mathrm{Conv}}_{3\times 3}\;(\mathrm{DA}(|{\hat{F}}_{s}-F_{f}|))+{\hat{F}}_{s},$$

ensuring that virtual-stain predictions (H&E, DAPI, LAP2, panCK) remain anchored to physics-constrained structural reconstructions rather than low-level intensity cues.

#### Joint End-to-End Optimization.

FPM2Stain Net is trained end-to-end with the combined loss:

(20) $$L_{\text{total}}=L_{\text{BiP-FPM}}+\lambda_{s}L_{\text{stain}},$$

where $L_{\text{stain}}$ includes pixel-wise $L_{1}/L_{2}$ , DP-IQA perceptual terms, and cross-task structural constraints. During early epochs, optical parameters (P,s) are frozen to stabilize pupil learning; later, all parameters are jointly optimized. This bidirectionally constrained strategy aligns super-resolution reconstruction and semantic colorization, yielding physically interpretable, high-fidelity virtual staining.

### 3.2. Reconstruction-to-staining interface and supervision

**Fig. 3.**
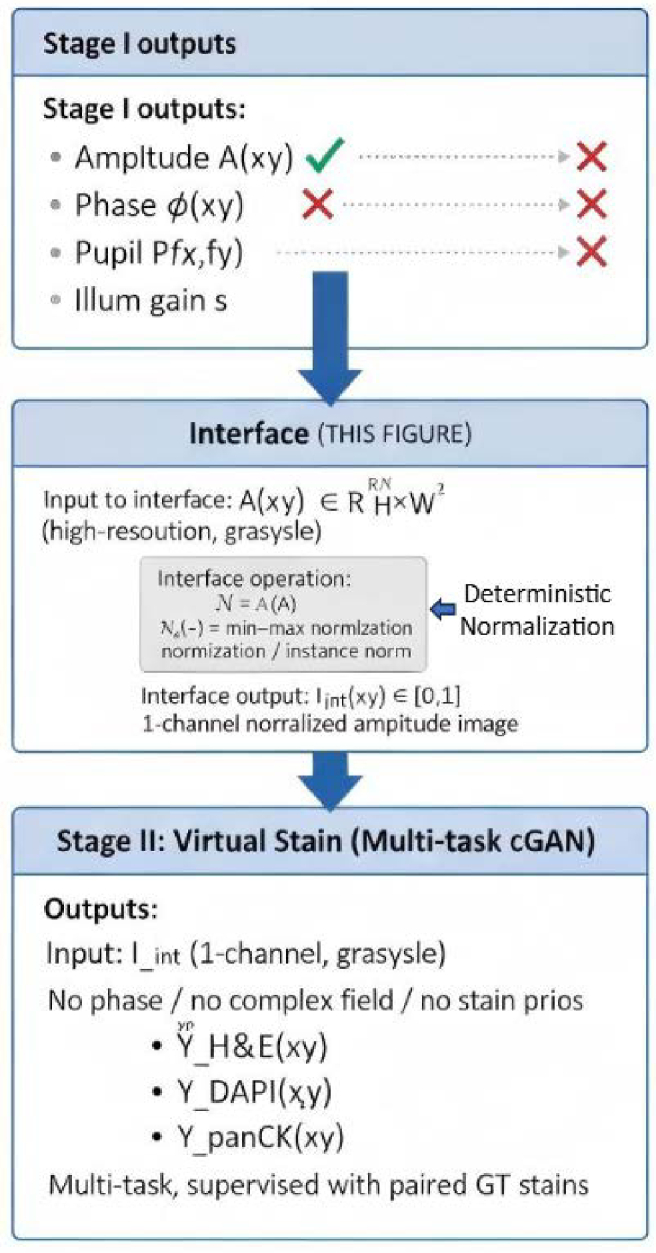
Interface between Stage I reconstruction and Stage II virtual staining. Stage I reconstructs a physically interpretable complex field and related system parameters; only the amplitude map A(x,y) is passed through a deterministic normalization N(⋅) to form the interface image $I_{\mathrm{int}}(x,y)\in [0,1]$ , The reconstructed amplitude image is fed into the staining network, where it serves as the only input to the multi-task virtual staining network. Phase, pupil, and illumination parameters are not forwarded to Stage II.

To address reproducibility and clearly define the interface between the reconstruction module (Stage I) and the virtual staining module (Stage II), we explicitly specify (i) the reconstruction outputs, (ii) the supervision used in the staining stage, and (iii) how color/style mismatch is mitigated. As summarized in Fig. 3, the staining module receives only the normalized reconstruction amplitude $I_{\mathrm{int}}=N(A)$ as input, while phase and system parameters are excluded from the Stage I→Stage II interface.

#### Stage I output (what the reconstruction module produces).

Given the raw FPM measurement(s) (multi-LED stack or single-shot capture, depending on the setting), the reconstruction module estimates physically interpretable quantities including the high-resolution complex field

(21) $${\hat{U}}_{HR}(x,y)=A(x,y)\exp(j\phi (x,y)),$$

together with optical/system parameters (e.g., pupil function *P* and illumination gain *s*). In our pipeline, the only tensor passed to Stage II is the reconstructed amplitude map A(x,y) (1-channel, grayscale). The phase map ϕ(x,y) is retained for physical validation/analysis but is not used as input to the staining network, avoiding potential instability and preventing any implicit modality leakage.

#### Interface tensor definition (exact input to staining).

We define the interface image as

(22) $$I_{\mathrm{int}}(x,y)=N\;\left(A(x,y)\right)\in [0,1],$$

where N(⋅) denotes a deterministic normalization (min–max per patch/field-of-view, or dataset-level normalization as specified in the implementation details). Stage II takes $I_{\mathrm{int}}$ as the sole image input, optionally followed by a lightweight intensity standardization layer (e.g., InstanceNorm or a 1×1 convolution) to reduce distribution shift across acquisitions.

#### Stage II supervision (what supervision the staining module uses).

The two stages employ fundamentally different training signals:

• Reconstruction (Stage I): trained self-supervised using physics-consistency between the observed measurement(s) and the differentiable forward model; no stained targets are used.
• Virtual staining (Stage II): trained paired-supervised with ground-truth staining targets for each modality (H&E, DAPI, LAP2, panCK). Specifically, for each training sample we use aligned pairs $(I_{\mathrm{int}},Y^{\left(c\right)})$ where $Y^{\left(c\right)}$ is the corresponding ground-truth stain channel/modal image for task *c*.

This strict separation ensures that Stage I never observes stain labels, while Stage II learns semantic colorization solely from $I_{\mathrm{int}}$ and the paired stain supervision.

#### Mitigating color/style mismatch (how inconsistency is handled).

To reduce color drift and inter-modality style mismatch, we combine complementary constraints:

1. Structural anchoring: all stain generators are conditioned on the same physics-constrained $I_{\mathrm{int}}$ , ensuring morphology-consistent colorization.
2. Perceptual/frequency consistency: we employ the DP-IQA perceptual supervision as a critic to suppress unrealistic textures and stabilize style.
3. Cross-task structural constraints: we impose biologically motivated constraints (e.g., nuclear consistency between DAPI and H&E; spatial exclusivity between LAP2 and panCK), discouraging spurious cross-channel co-activation and reducing modality-specific style artifacts.

Together, these design choices make the Stage I → Stage II interface explicit and reproducible, while improving robustness against color/style mismatch.

### 3.3. Multi-task staining model

Building upon the physics-constrained reconstruction of BiP-FPM, the second stage of FPM2Stain Net performs multi-task virtual staining, guided by physics-constrained structural priors from BiP-FPM. Instead of relying solely on data-driven translation, the staining network receives structurally rich, physically consistent features extracted from the BiP-FPM encoder. This design ensures that each synthesized stain—H&E, DAPI, LAP2, and panCK—is grounded in optically faithful morphology rather than heuristic intensity patterns.

The multi-task staining model adopts an encoder–fusion–decoder architecture that jointly predicts multiple staining modalities. It integrates (1) a spatial branch initialized by BiP-FPM features, (2) a frequency branch based on wavelet transform convolution (WTConv), and (3) a frequency–spatial feature fusion (FS-FF) module with attention-guided integration. Perceptual quality and biological consistency are further enhanced through a Difference-Perception Image Quality Assessment (DP-IQA) critic and cross-task structural constraints.

#### Encoder with Dual-Branch Feature Extraction.

The encoder comprises a shallow convolutional stem (Conv 3×3 + ReLU) followed by two down-sampling blocks. To ensure structural coherence, the spatial branch is directly initialized by the BiP-FPM encoder’s multi-scale feature maps $\left\{F_{s}^{i}\right\}$ rather than re-learned from raw intensities. Each feature map is resized and channel-aligned via 1×1 convolutions:

(23) $${\hat{F}}_{s}^{i}={\mathrm{Conv}}_{1\times 1}(\mathrm{Resize}(F_{s}^{i})),i=1,2,3.$$

In parallel, the frequency branch employs wavelet transform convolution (WTConv) to decompose features into four sub-bands (LL, LH, HL, HH), capturing texture and edge information across scales. Both branches are fused in the FS-FF module described below.

**Fig. 4.**
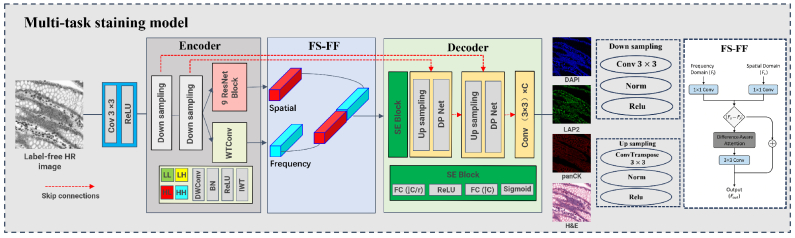
Overview of the proposed multi-task staining model. The network integrates spatial and frequency features via a dual-branch encoder and a frequency–spatial feature fusion module (FS-FF), followed by a decoder with SE attention and DP-IQA supervision to jointly predict LAP2, DAPI, panCK, and H&E stainings.

We propose a novel multi-task virtual staining network that learns to map high-resolution label-free microscopic images to multiple biologically relevant staining domains, including LAP2, DAPI, panCK, and H&E. As illustrated in Fig. 4, the overall architecture follows an encoder–fusion–decoder design paradigm, integrating frequency–spatial feature fusion module (FS-FF), channel-wise attention (Squeeze-and-Excitation (SE) block), and cross-task structural constraints to ensure consistency, precision, and biological plausibility across all staining modalities.

#### Encoder with Dual-Branch Feature Extraction.

The encoder consists of a shallow convolutional stem (Conv 3×3 + ReLU), followed by two down-sampling blocks composed of convolution, normalization, and ReLU activation. This is followed by two parallel feature extraction branches:

• Spatial Branch: Unlike conventional staining encoders, the spatial branch is directly replaced by multi-scale features $\left\{{\hat{F}}_{s}^{i}\right\}$ extracted from the 9-ResNet encoder of BiP-FPM, after resize and 1×1 projection. The fused representation is then integrated through the FS-FF module, ensuring that stain predictions are anchored to super-resolved structures rather than low-level intensity cues.
• Frequency Branch: A Wavelet Transform Convolution (WTConv) module that decomposes the feature maps into four subbands (LL, LH, HL, HH), processed by depthwise convolutions and reconstructed via inverse wavelet transform (IWT). This branch enhances high-frequency components such as edges and textures.

#### Frequency–Spatial Feature Fusion Module (FS-FF).

The Frequency–Spatial Feature Fusion Module (FS-FF) integrates the spatial features $F_{s}$ and frequency features $F_{f}$ by projecting them through 1×1 convolutions followed by a difference-aware attention mechanism:

(24) $$F_{\text{out}}={\text{Conv}}_{3\times 3}\;\left(\text{DA}\left(|{\hat{F}}_{s}-F_{f}|\right)\right)+{\hat{F}}_{s},$$

where DA denotes a light-weight difference-aware attention module. The fused feature map $F_{\text{out}}$ is passed to the decoder for multi-task generation.

#### Decoder with Channel Attention and Difference-Perception Supervision.

The decoder progressively restores spatial resolution through transposed convolutions, batch normalization, and ReLU activations. At each upsampling stage, a SE block is applied to adaptively recalibrate channel-wise feature responses, emphasizing task-relevant stain characteristics. To further preserve fine-grained spatial details, skip connections from the encoder are fused at corresponding scales.

**Fig. 5.**
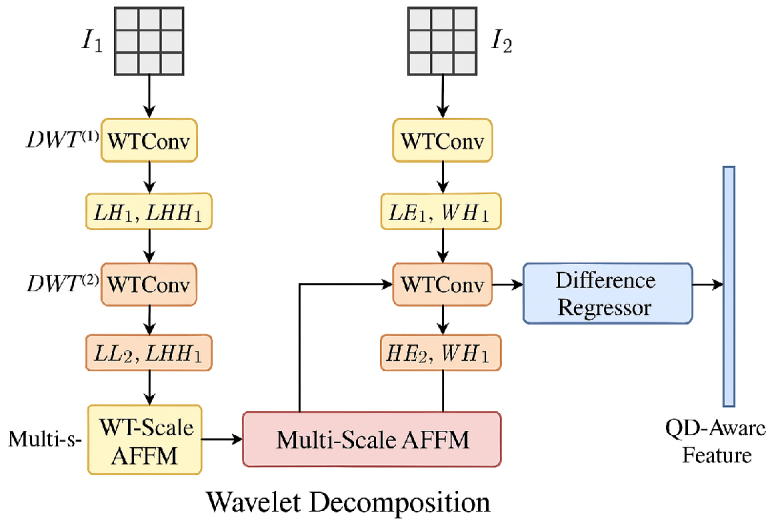
Architecture of the Difference-Perception Network (DP Net) in DP-IQA. Two inputs $I_{1}$ and $I_{2}$ are processed by multi-level DWT and WTConv to extract spatial–frequency features. A multi-scale asymmetric feature fusion module (MS-AFFM) aggregates features across levels, and a difference regressor predicts perceptual dissimilarity, yielding quality-difference–aware (QD-aware) features.

In parallel, a Difference-Perception IQA (DP-IQA) supervision module is embedded at multiple resolution levels to enforce perceptual fidelity (Fig. 5). DP-IQA is a dual-branch, wavelet-based network originally designed for no-reference image quality assessment. It consists of:

• Main regression branch: A deep convolutional encoder (ResNet-50) extracts semantic and structural features, followed by global average pooling and optional channel attention to produce a compact semantic vector. A multilayer perceptron (MLP) regresses an absolute image quality score ${\hat{q}}_{\mathrm{main}}$ .
• Difference-aware auxiliary branch: Models perceptual differences between image pairs $\left(I_{1},I_{2}\right)$ via two-level discrete wavelet transforms (DWT), wavelet convolutions (WTConv), and a Multi-Scale Asymmetric Feature Fusion Module (MS-AFFM). The auxiliary branch outputs a difference-aware representation $F_{\mathrm{diff}}$ and a predicted perceptual difference $\hat{d}$ , which are also injected into the main branch to refine ${\hat{q}}_{\mathrm{main}}$ .

MS-AFFM aligns features spatially, applies asymmetric attention $\alpha_{s}=\sigma (W_{2}\delta (W_{1}\mathrm{GAP}(F_{s})))$ , fuses them across scales $F_{\mathrm{fused}}=\sum_{s}\alpha_{s}⊙F_{s}$ , and performs residual enhancement.

The DP-IQA training objective combines absolute and relative quality supervision:

(25) $$L_{\mathrm{IQA}}=L_{\mathrm{main}}+\lambda_{1}L_{\mathrm{aux}}+\lambda_{2}L_{\mathrm{diff}},$$

where $L_{\mathrm{main}}={∥{\hat{q}}_{\mathrm{main}}-q∥}_{1},L_{\mathrm{aux}}={∥{\hat{q}}_{\mathrm{aux}}-q∥}_{1}$ , and $L_{\mathrm{diff}}={∥\hat{d}-|q_{1}-q_{2}|∥}_{1}$ .

During FPM2Stain Net training, DP-IQA acts as a perceptual critic at multiple resolution stages, encouraging the generator to preserve fine-grained structural cues and frequency details that are critical for human-perceived image quality. This perceptual guidance complements pixel-wise $L_{1}$ and cross-task structural constraints, resulting in stain outputs that are both numerically accurate and visually faithful.

The final decoder output is a multi-channel tensor of size H×W×C , where C=4 corresponds to the predicted LAP2, DAPI, panCK, and H&E staining maps, respectively.

Cross-Task Constraint Loss. To enforce biologically plausible relationships across the predicted stain modalities, we introduce a Cross-Task Structural Constraint Loss $L_{\text{cross-task}}$ . This loss explicitly models the structural dependencies and exclusivities among staining outputs (LAP2, DAPI, panCK, and H&E), based on three types of constraints:

##### (1) Structural Consistency Loss

DAPI and H&E images share common nuclear structures. We encourage the predicted DAPI and H&E outputs to align within nuclear regions by computing a masked mean squared error (MSE):

(26) $$L_{\text{consistency}}={\left\|M_{\text{nuc}}\cdot \left({\hat{y}}_{\text{DAPI}}-{\hat{y}}_{\text{H\&E}}\right)\right\|}_{2}^{2}$$

where $M_{\text{nuc}}$ is a binary nuclear mask derived from thresholded DAPI predictions.

##### (2) Structural Exclusivity Loss

LAP2 and panCK are known to mark mutually exclusive structures (e.g., nuclear envelope vs. cytokeratin). We penalize their spatial overlap using an element-wise product:

(27) $$L_{\text{exclusive}}=\text{mean}\left({\hat{y}}_{\text{LAP2}}\cdot {\hat{y}}_{\text{panCK}}\right)$$

This discourages spatial co-activation between biologically distinct stainings.

##### (3) Relative Attention Consistency Loss

To align task attention across modalities, we use DAPI as an anchor and minimize the discrepancy in attention maps between DAPI and other outputs (e.g., LAP2 or panCK):

(28) $$L_{\text{relative}}={\left\|\text{Attn}\left({\hat{y}}_{\text{DAPI}}\right)-\text{Attn}\left({\hat{y}}_{\text{target}}\right)\right\|}_{1}$$

where Attn(⋅) denotes a spatial attention map extracted using either CAM, soft attention pooling, or learned feature activations.

#### Total Cross-Task Loss

The overall cross-task constraint is defined as a weighted combination:

(29) $$L_{\text{cross-task}}=\alpha \cdot L_{\text{consistency}}+\beta \cdot L_{\text{exclusive}}+\gamma \cdot L_{\text{relative}}$$

where α,β,γ are empirically set balancing coefficients.

This loss is integrated into the total objective as:

(30) $$L_{\text{stain}}=\sum_{c=1}^{4}\left(L_{\text{GAN}}^{c}+\lambda_{1}L_{\text{L1}}^{c}+\lambda_{2}L_{\text{DP-IQA}}^{c}\right)+\lambda_{3}L_{\text{cross-task}}$$

This multi-term objective loss function encourages each modality to be faithfully predicted while maintaining semantic consistency across modalities.

## 4. Experiments

### 4.1. Experimental setup

The experiments consist of three parts. First, the BiP-FPM module is evaluated using the standard Baboon image (for simulated FPM reconstruction) and the real USAF resolution dataset collected by Tomas Aidukas [59], to verify the model’s capability to recover high-resolution amplitude and phase from low-resolution LED images. Second, we assess the Multi-task Staining Model independently using the HEMIT [60] and DeepLIIF [56] histological datasets, where high-resolution grayscale structural inputs are translated into multiple stains (DAPI, LAP2, panCK, H&E) and compared to ground truth. Finally, to evaluate the full FPM2Stain Net pipeline, we simulate FPM-style degradation on HEMIT and DeepLIIF data, feeding the resulting low-resolution label-free images into the end-to-end system to jointly test reconstruction and staining performance. We further perform downstream tasks, Cellpose-based cell segmentation [61] and fluorescence intensity quantification, to assess the utility of the synthesized stains in computational pathology tasks. All datasets are divided into non-overlapping splits of 70% for training, 15% for validation, and 15% for testing. To ensure independence between training and testing, datasets are partitioned at the level of tissue slides or patients, such that no sample from the same specimen or subject appears in both splits. All quantitative metrics (PSNR, SSIM, DP-IQA, segmentation mIoU) are reported on the independent test sets to avoid overfitting bias.

#### USAF Resolution Target.

To further validate the reconstruction capacity of BiP-FPM, we conducted experiments on a USAF resolution chart using the low-cost FPM setup described in [59]. The hardware consists of a Raspberry Pi V2 NOIR camera module (8 MP, 1.12 *μ*m pixel size) with a remounted 3-mm focal-length lens providing ∼1.5× magnification, combined with a 16×16 RGB LED array (3.3-mm pitch) positioned 60 mm below the sample. This configuration provides 0.15 NA objective imaging with a synthetic illumination NA of 0.4, yielding an effective synthetic NA of 0.55. At λ=470nm (blue LED), this corresponds to a theoretical resolution of ∼780 nm. A total of 256 raw low-resolution images were captured under sequential LED illuminations with adaptive exposure times to optimize SNR. These conditions follow the protocol described in [59] and allow for a direct comparison of reconstruction quality.

#### Datasets and Data Splits.

We evaluated FPM2Stain Net on three complementary data sources: (1) the HEMIT dataset [60], containing H&E histology images paired with multiplex immunofluorescence (mpIF) channels; (2) the DeepLIIF dataset [56], which provides large-scale immunofluorescence tissue images; and (3) synthetically degraded FPM-style data generated from HEMIT and DeepLIIF by applying the forward model with Zernike-modeled PSFs.

### 4.2. Implementation details

All models were implemented in PyTorch 2.0 and trained on an NVIDIA RTX 4090 GPU with 24 GB of memory. The BiP-FPM module employs a ResNet-U-Net backbone with nine residual blocks to reconstruct 512×512 amplitude and phase maps from 256×256 multi-LED FPM intensity inputs. It is optimized using the Adam optimizer $\left(\text{lr}=2\times 10^{-4}\right)$ for 200 epochs under a self-supervised loss comprising total-variation and pupil regularization terms $\left(\lambda_{\mathrm{tv}}=10^{-4},\lambda_{P}=10^{-3}\right)$ .

The Multi-task Staining Model takes the reconstructed amplitude map as input and employs a ResNet-9 encoder, WTConv-based frequency branch, FS-FF fusion module, and SE-enhanced decoder. It generates four stain modalities (DAPI, LAP2, panCK, and H&E) and is trained using a composite objective with L1, DP-IQA, and cross-task constraint losses. The loss weights are set as $\lambda_{1}=10.0,\lambda_{2}=0.2$ , and $\lambda_{3}=1.0$ , with cross-task sub-weights α=1.0,β=0.5 , and γ=0.2 . A PatchGAN discriminator with a 70×70 receptive field is applied independently to each output stain and trained with a least-squares GAN (LSGAN) objective to enhance local texture realism. Training uses Adam $\left(\text{lr}=1\times 10^{-4}\right)$ , batch size 8, and 150 epochs with standard data augmentation (random flipping, rotation, and brightness jittering). Joint Training Hyperparameters. For the full end-to-end pipeline, the overall objective is defined as:

(31) $$L_{\text{total}}=L_{\text{BiP-FPM}}+\lambda_{s}L_{\text{stain}},L_{\text{BiP-FPM}}=L_{\text{phys}}+\lambda_{d}L_{\text{deconv}}.$$

In our experiments, $\lambda_{s}=0.5$ balances reconstruction fidelity and staining accuracy, and $\lambda_{d}=0.1$ provides stable contribution from the PSF-based deconvolution constraint. A warm-up schedule linearly increases $\lambda_{s}$ from 0.1 to 0.5 during the first 10 epochs, ensuring early optimization is dominated by the physics-consistency loss $L_{\text{BiP-FPM}}$ , while gradually allowing $L_{\text{stain}}$ to refine semantic colorization. All other hyperparameters, including optimizer settings and data augmentations, follow the same configuration as described for the individual modules.

### 4.3. BiP-FPM module feasibility verification

#### 4.3.1. Motivation of the PSF deconvolution network

The proposed architecture consists of two complementary components: a physics-based Fourier ptychographic reconstruction network (pbNN) and a PSF deconvolution network. The pbNN layer aims to recover a high-resolution complex-valued image from multiplexed low-resolution measurements by learning an inverse mapping guided by the FPM forward model. However, due to residual optical blur and model mismatch, the pbNN output may still deviate from a physically consistent reconstruction.

To address this limitation, a PSF deconvolution network is introduced as a second stage to explicitly enforce optical consistency. Rather than “recorrupting” the reconstruction, this module refines the intermediate result by suppressing residual blur and constraining the spatial frequency response according to the estimated point spread function (PSF). The output of this stage is therefore referred to as a *PSF-consistent reconstruction*.

To justify the necessity of the proposed two-stage architecture, an ablation study was conducted by comparing the following configurations:

• pbNN-only: using only the physics-based reconstruction network;
• PSF-only: applying PSF deconvolution without pbNN reconstruction;
• pbNN + PSF (Full): the complete proposed framework.

Quantitative results in terms of PSNR, SSIM, and contrast demonstrate that removing the PSF deconvolution layer consistently degrades reconstruction quality. These findings confirm that the PSF layer plays a critical role in refining the pbNN output and improving both reconstruction fidelity and downstream virtual staining performance.

**Fig. 6.**
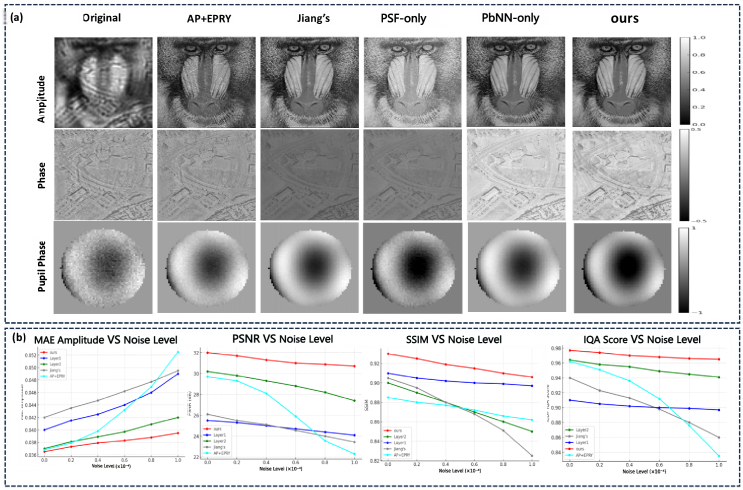
Comparison of FPM reconstruction results with sample of baboon for the original input low resolution image, AP+EPRY, Jiang’s PbNN baseline, our ablated PbNN (PSF-only/PbNN-only), and the proposed BiP-FPM model under various conditions (Gaussian distribution noises with zero mean and SD of zero to $1\times 10^{-4}$ ).

To evaluate the robustness and generalization ability of the BiP-FPM module, the experiment used reconstructed images of a baboon and USAF target from a public test dataset to verify the reconstruction performance. The relevant parameters in the Baboon target were set as follows: the magnification was set to 4 times, the NA to 0.13, the pixel size to 6.5*μ*m, the illumination wavelength to 505 nm, the 13 x 13 LED array was placed 98 mm below the specimen, and the distance between adjacent LEDs was 8 mm. To assess the network’s robustness to imaging noise, we compared the reconstruction results of different methods under various conditions (Gaussian distribution noise with zero mean and a standard deviation of zero to $1\times 10^{-4}$ ). As shown in Fig. 6 and Table 1, the proposed BiP-FPM consistently outperformed all baseline and ablated models under increasing levels of synthetic noise. Specifically, it achieved the lowest amplitude MAE and the highest PSNR, SSIM, and DP-IQA scores across all noise conditions. The degradation trends were significantly slower compared to models without Zernike-based pupil modeling (PSF-only) or without PSF physics-based supervision (PbNN-only), indicating that both components are critical for stabilizing the reconstruction process. These results validate the robustness of BiP-FPM and demonstrate its potential for real-world imaging scenarios where measurement noise is inevitable.

**Table 1.** Noise-robustness on Baboon test set. For each noise level $\left(\times 10^{-4}\right)$ , N=100 non-overlapping patches/fields were sampled. We report mean±SD of MAE, PSNR, SSIM, and DP-IQA on the held-out test set. Best results for each noise level are shown in bold.

Metric	Method	0.0	0.2	0.4	0.6	0.8	1.0
MAE ↓	Ours	**0.036**±0.002	**0.037**±0.002	**0.038**±0.003	**0.039**±0.003	**0.040**±0.004	**0.041**±0.004
	PSF-only	0.041±0.003	0.043±0.003	0.045±0.004	0.046±0.004	0.047±0.004	0.048±0.005
	PbNN-only	0.037±0.002	0.038±0.003	0.039±0.003	0.040±0.003	0.041±0.004	0.042±0.004
	Jiang’s	0.040±0.003	0.042±0.003	0.044±0.004	0.046±0.004	0.048±0.005	0.049±0.005
	AP+EPRY	0.037±0.003	0.039±0.004	0.042±0.004	0.045±0.005	0.049±0.005	0.053±0.006

PSNR ↑ (dB)	Ours	**32.02**±0.15	**31.81**±0.16	**31.73**±0.18	**31.25**±0.19	**30.93**±0.20	**30.74**±0.22
	PSF-only	25.56±0.25	25.30±0.28	24.97±0.30	24.64±0.32	24.32±0.34	24.03±0.36
	PbNN-only	30.01±0.18	29.54±0.20	29.03±0.22	28.52±0.23	28.01±0.25	27.52±0.27
	Jiang’s	29.54±0.21	29.01±0.23	28.56±0.25	28.05±0.26	27.53±0.28	27.02±0.30
	AP+EPRY	29.62±0.20	29.37±0.22	28.36±0.24	25.96±0.25	23.89±0.27	22.27±0.29

SSIM ↑	Ours	**0.922**±0.006	**0.919**±0.006	**0.916**±0.007	**0.913**±0.007	**0.911**±0.007	**0.909**±0.008
	PbNN-only	0.910±0.007	0.908±0.007	0.906±0.007	0.904±0.007	0.902±0.008	0.900±0.008
	PSF-only	0.900±0.008	0.898±0.008	0.896±0.008	0.894±0.009	0.892±0.009	0.890±0.009
	Jiang’s	0.888±0.009	0.882±0.009	0.876±0.010	0.870±0.010	0.865±0.010	0.860±0.011
	AP+EPRY	0.885±0.009	0.882±0.009	0.879±0.010	0.875±0.010	0.871±0.011	0.868±0.011

DP-IQA ↑	Ours	**0.978**±0.004	**0.976**±0.004	**0.974**±0.005	**0.972**±0.005	**0.970**±0.005	**0.968**±0.006
	PbNN-only	0.965±0.005	0.963±0.005	0.961±0.005	0.959±0.006	0.957±0.006	0.955±0.006
	PSF-only	0.920±0.007	0.918±0.007	0.916±0.007	0.914±0.008	0.912±0.008	0.910±0.008
	Jiang’s	0.940±0.006	0.930±0.006	0.920±0.007	0.910±0.007	0.900±0.008	0.890±0.008
	AP+EPRY	0.960±0.005	0.950±0.006	0.940±0.006	0.920±0.007	0.900±0.007	0.860±0.008

**Fig. 7.**
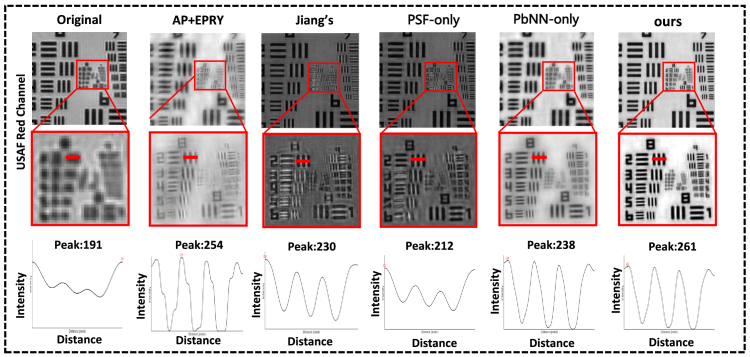
USAF resolution target and intensity line profiles from the red channel are shown for the original input low resolution image, AP+EPRY, Jiang’s PbNN baseline, our ablated PbNN (PSF-only/PbNN-only), and the proposed BiP-FPM model. The proposed approach preserves sharper peaks (e.g., 191–261) and higher contrast compared to competing methods, demonstrating improved resolution recovery and robustness to system aberrations.

To further assess the robustness and generalization ability of the proposed BiP-FPM module, we conducted reconstruction experiments using the USAF resolution chart. Unlike conventional FPM pipelines that require multiple sequentially illuminated acquisitions, our BiP-FPM is designed to recover high-resolution amplitude and phase from *a single brightfield image*. In practice, we verified that arbitrary single-frame brightfield captures of the USAF target can be directly used as inputs to BiP-FPM, demonstrating the capability of single-shot reconstruction without relying on coded or multiplexed illumination. As shown in Fig. 7, the reconstructed results show that BiP-FPM achieves ∼780 nm effective resolution (corresponding to group 10), consistent with a synthetic NA of 0.55, and preserves sharp peaks in the red-channel intensity line profiles. This confirms that the physics-guided BiP-FPM front-end not only generalizes to real optical hardware with low-cost components but also maintains the ability to reconstruct from individual label-free brightfield measurements, highlighting its potential for practical single-shot microscopy. Figure 7 and Fig. 6 illustrates the progressive effect of stacking pbNN and PSF layers, while Table 1 provides a controlled ablation demonstrating that removing the PSF layer leads to consistent degradation.

### 4.4. Digital staining module feasibility verification

To evaluate the effectiveness and generalizability of the proposed digital staining module across data sets, we performed training and testing on the HEMIT colorectal tissue dataset [60] and lung/bladder tissue samples from the DeepLIIF study [56]. The target staining types include H&E, DAPI, LAP2, and panCK. Our method was benchmarked against current state-of-the-art (SOTA) approaches, including UNet, ResNet-9blocks [56], four pix2pix-based models (pix2pix_ResNet [47], pix2pix_UNet [43,54], pix2pix_CycleGAN [47],pix2pix_Transformer [60]), and the diffusion-based DiffuStain [42]. Systematic experiments across diverse tissue types and staining tasks were conducted to assess the model’s performance in terms of staining fidelity, multi-task generation capability, and generalization across datasets. Quantitative comparisons between FPM2Strain Net and representative baseline methods are summarized in Table 2.

**Table 2.** Comparison of quantitative performance and computational efficiency across different methods and tissue types. Inference time is reported as the average seconds per image under identical hardware settings.

**Colorectal Marker (DAPI H&E panCK)**														

Tissue	Methods	SSIM				DP-IQA				PSNR (dB)				Time (s)

		DAPI	H&E	panCK	Avg	DAPI	H&E	panCK	Avg	DAPI	H&E	panCK	Avg	

Colorectal	U-Net	0.791	0.877	0.891	0.866	0.659	0.805	0.919	0.794	27.929	24.733	30.773	27.812	**1.85**
	ResNet-9blocks [56]	0.790	0.907	0.868	0.855	0.877	0.785	0.919	0.860	27.531	24.533	30.212	27.425	2.35
	pix2pix_ResNet [47]	0.775	0.879	0.913	0.852	0.755	0.833	0.952	0.849	27.691	25.659	34.306	29.219	2.28
	pix2pix_UNet [43,54]	0.786	0.900	0.908	0.865	0.821	0.856	0.923	0.867	28.156	26.281	32.359	28.932	2.10
	pix2pix_CycleGAN [47]	0.816	0.915	0.911	0.881	0.826	0.880	0.941	0.882	30.276	28.521	34.039	30.945	2.40
	pix2pix_Transformer [60]	0.815	0.910	0.913	0.879	0.896	0.877	0.939	0.904	29.276	**28.610**	34.875	30.920	2.95
	DiffuStain [42]	0.823	0.906	**0.914**	0.881	0.923	0.853	0.946	0.907	**33.152**	25.223	34.359	30.911	17.80
	**Ours**	**0.833**	**0.918**	0.911	**0.887**	**0.938**	**0.903**	**0.954**	**0.932**	32.395	28.349	**35.145**	**31.963**	3.32

**Lung Marker (DAPI H&E LAP2)**														
Tissue	Methods	SSIM				DP-IQA				PSNR (dB)				Time (s)

		DAPI	H&E	LAP2	Avg	DAPI	H&E	LAP2	Avg	DAPI	H&E	LAP2	Avg	

Lung	U-Net	0.799	0.916	0.910	0.875	0.669	0.813	0.958	0.812	28.208	24.980	34.111	29.100	**1.90**
	ResNet-9blocks [56]	0.828	0.916	0.909	0.884	0.886	0.793	0.958	0.879	30.806	27.778	34.544	31.043	2.38
	pix2pix_ResNet [47]	0.783	0.888	0.912	0.861	0.763	0.841	0.957	0.853	27.968	25.916	34.649	29.511	2.35
	pix2pix_UNet [43,54]	0.794	0.909	0.917	0.873	0.829	0.865	0.932	0.876	28.438	26.544	32.683	29.222	2.11
	pix2pix_CycleGAN [47]	0.824	0.924	0.920	0.889	0.834	0.889	0.950	0.891	**33.579**	28.806	34.379	32.255	2.43
	pix2pix_Transformer [60]	0.845	0.917	0.919	0.894	0.906	0.885	0.959	0.917	32.336	28.570	34.561	31.822	2.98
	DiffuStain [42]	0.854	0.935	**0.923**	0.904	0.932	0.862	0.955	0.916	33.484	25.475	34.703	31.220	18.10
	**Ours**	**0.873**	**0.937**	0.913	**0.908**	**0.938**	**0.933**	**0.971**	**0.947**	32.311	**29.349**	**35.145**	**33.268**	3.33
**Bladder Marker (DAPI H&E LAP2)**														

Tissue	Methods	SSIM				DP-IQA				PSNR (dB)				Time (s)

		DAPI	H&E	LAP2	Avg	DAPI	H&E	LAP2	Avg	DAPI	H&E	LAP2	Avg	

Bladder	U-Net	0.807	0.925	0.919	0.884	0.672	0.821	0.968	0.820	28.488	25.228	34.448	29.388	**1.88**
	ResNet-9blocks [56]	0.806	0.925	0.916	0.882	0.895	0.801	0.968	0.887	28.082	25.024	33.876	28.994	2.41
	pix2pix_ResNet [47]	0.791	0.897	0.921	0.869	0.770	0.850	0.967	0.862	28.245	26.172	34.992	29.803	2.31
	pix2pix_UNet [43,54]	0.802	0.918	0.926	0.882	0.837	0.873	0.941	0.884	28.719	26.807	33.006	29.511	2.08
	pix2pix_CycleGAN [47]	0.832	**0.933**	0.929	0.896	0.843	0.898	0.960	0.900	**33.882**	28.916	34.720	32.506	2.25
	pix2pix_Transformer [60]	0.835	0.930	**0.933**	0.899	0.905	0.886	0.948	0.913	32.556	28.718	34.991	32.088	3.01
	DiffuStain [42]	0.862	0.924	0.932	0.906	0.941	0.870	0.965	0.925	33.815	28.727	35.046	32.529	16.89
	**Ours**	**0.887**	0.929	0.932	**0.916**	**0.952**	**0.891**	**0.980**	**0.941**	32.937	**29.391**	**35.888**	**32.739**	3.15

**Fig. 8.**
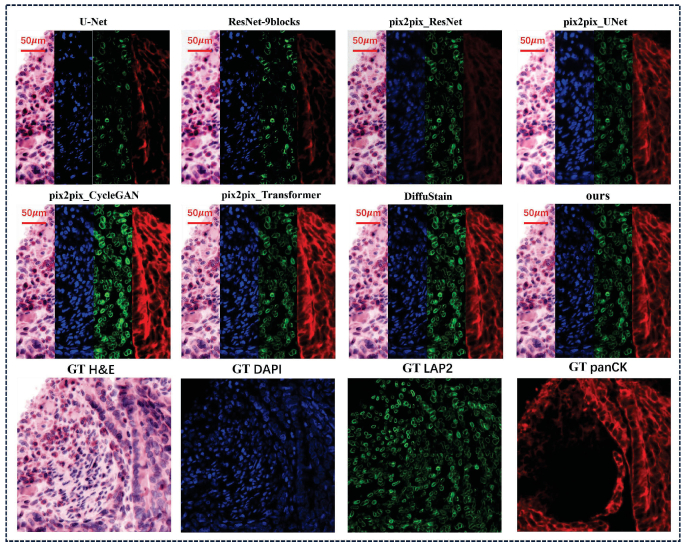
Comparison of digital staining results across multiple methods. Each column shows the digitally generated staining results (H&E, DAPI, LAP2, panCK) for the same tissue region using different methods. All images are aligned to the same field of view.

Although the proposed method achieves pixel-wise metrics comparable to the diffusion-based DiffuStain model, the superiority of FPM2Stain Net should not be interpreted solely in terms of PSNR or SSIM. As illustrated in Fig. 8, the visual differences between the two methods can appear subtle; however, pixel-level visual realism alone does not fully reflect practical performance in computational pathology. Instead, our advantage lies in dimensions that are critical for real-world deployment but are not fully captured by conventional image fidelity measures. Specifically, FPM2Stain Net enforces cross-modality structural consistency by jointly predicting multiple staining channels under biologically motivated constraints, leading to more coherent multi-stain representations. Moreover, by decoupling physics-guided reconstruction from semantic staining, the generated outputs are anchored to physically interpretable optical structures rather than learned statistical priors alone. Importantly, our method is substantially more computationally efficient: the full FPM2Stain Net pipeline requires over five times less inference time than DiffuStain under identical hardware settings, making it considerably more suitable for high-throughput and time-sensitive microscopy workflows. This combination of structural consistency, physical interpretability, downstream stability, and computational efficiency establishes FPM2Stain Net as a more practical alternative for multi-modal virtual staining from FPM data.

### 4.5. Downstream analysis of FPM2Stain Net Results

**Fig. 9.**
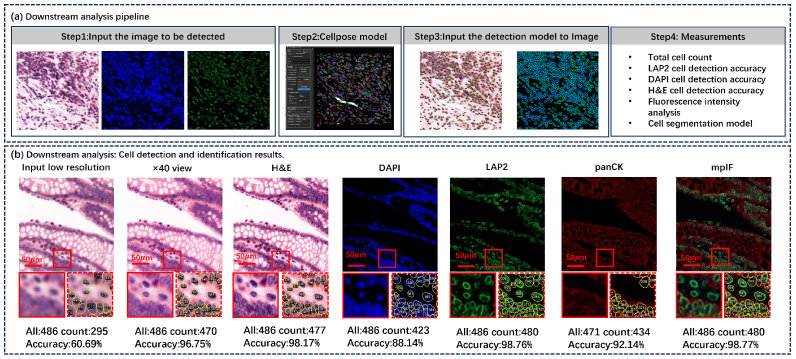
Downstream analysis enabled by FPM2Stain Net-generated virtual stains. Top: Workflow using Cellpose on virtual stains (H&E, DAPI, LAP2) generated by FPM2Stain Net. Bottom: FPM2Stain Net-stained images enable accurate cell segmentation across channels. Notably, virtual H&E outperforms images acquired using a conventional 40× objective microscope in segmentation precision, demonstrating a pixel-level upsampling factor exceeding 10×.

**Fig. 10.**
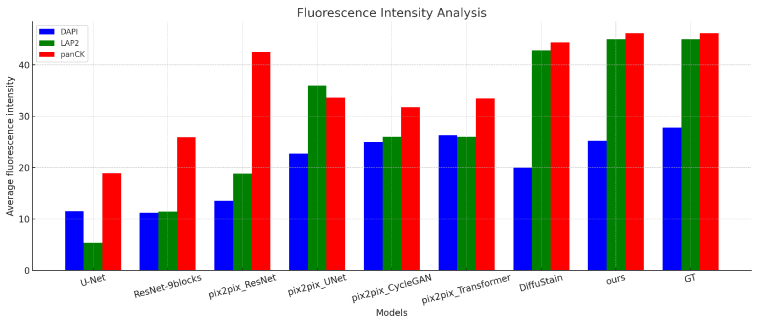
Fluorescence intensity distributions of DAPI, LAP2, and panCK channels across different methods, compared against ground-truth (GT) staining. Virtual stains generated by FPM2Stain Net exhibit the closest alignment with GT, confirming both structural fidelity and biological interpretability

To evaluate the clinical potential of FPM2Stain Net, we conducted a cell detection experiment using the Cellpose model and analyzed fluorescence intensity with ImageJ, as shown in Fig. 9 and Fig. 10. As illustrated in the top panel, the pipeline includes: (1) inputting the predicted stained images; (2) applying the Cellpose model [61] for nucleus/cell segmentation; (3) mapping the segmentation masks back to each channel; and (4) enabling measurement of cell-level morphology and spatial distribution.

The bottom panel presents segmentation outcomes on each digitally stained channel, including close-up views with numbered cell boundaries. Compared to the original label-free input, the synthesized stains significantly enhance nuclear contrast (DAPI), membrane clarity (LAP2), and overall structural definition (H&E, panCK), facilitating accurate cell contour delineation and quantification. FPM2Stain Net’s H&E reconstruction achieved a recognition accuracy of 98.17%, surpassing the 96.75% of a 40× microscope, with complete staining accuracy of reaching 95%. Notably, the H&E images generated by FPM2Stain Net achieved higher cell segmentation accuracy than images acquired using a conventional 40× objective microscope when processed with the Cellpose model. This result indicates that FPM2Stain Net successfully achieves a pixel-level upsampling factor exceeding 10× , enabling high-fidelity virtual staining with enhanced spatial detail.

These results validate that FPM2Stain Net not only generates visually realistic and diagnostically informative staining outputs but also produces features highly compatible with downstream computational pathology tools, supporting tasks such as cell counting, phenotyping, and tissue structure analysis with exceptional resolution and accuracy.

To further quantify staining fidelity, we analyzed the average fluorescence intensity of DAPI, LAP2, and panCK across models (Fig. 10). FPM2Stain Net consistently achieved the highest or near-highest intensity values in all channels, outperforming both pix2pix-based and diffusion-based baselines. This indicates not only superior signal reconstruction but also biologically meaningful staining that supports robust downstream analysis.

Beyond standalone validation, the proposed FPM2Stain Net can be seamlessly embedded into routine clinical pathology pipelines. In a typical workflow, unstained tissue sections can be directly imaged at low magnification (4×) using a cost-effective FPM setup. FPM2Stain Net then automatically processes the resulting single-shot acquisitions to generate virtual H&E and multiplex immunofluorescence channels in under four seconds per field of view, eliminating the need for chemical staining or multi-round immunolabeling. The digitally stained outputs are fully compatible with standard pathology viewers (e.g., ImageJ, QuPath) and can be directly subjected to downstream computational pathology tasks such as cell segmentation, biomarker quantification, or tumor-immune microenvironment analysis. This integration enables near real-time turnaround for histological examination, reduces reagent and labor costs, and preserves tissue integrity for additional molecular assays. Importantly, the plug-in style deployment of the virtual staining module (e.g., via ImageJ toolbox extension) can facilitate rapid adoption in the existing digital pathology infrastructures, paving the way for multi-center clinical validation and point-of-care diagnostics.

### 4.6. Ablation studies

To investigate the contribution of each key component in the proposed multi-task staining model, we conducted a series of ablation experiments by progressively removing or altering specific architectural modules. Table 3 summarizes the quantitative performances of six model variants evaluated on multiple metrics: SSIM for H&E images, average fluorescence intensity for DAPI, LAP2, and panCK channels, Cellpose-based segmentation accuracy (mIoU), and inference time per image.

**Table 3.** Quantitative results of ablation study on the multi-task staining model. Metrics include SSIM for H&E images, average fluorescence intensity for DAPI, LAP2, and panCK channels, and mIoU for Cellpose-based segmentation accuracy. Two additional ablations are added: whether the pipeline is trained end-to-end (*w/o Joint Training*) and whether BiP-FPM spatial features are fused into the staining module (*w/o BiP-FPM-Feat* vs. *BiP-FPM-Feat (ours)*).

Variant	H&E SSIM ↑	DAPI Intensity ↑	LAP2 Intensity ↑	panCK Intensity ↑	Seg. mIoU ↑	Time (s) ↓
Full (Ours)	0.928	22.8	38.1	45.3	0.812	3.32
w/o WTConv	0.849	17.2	29.4	38.5	0.743	3.21
w/o FS-FF	0.872	15.6	27.0	35.9	0.712	4.63
w/o SE block	0.904	19.3	33.5	40.2	0.768	3.74
w/o DP Net	0.852	14.6	25.1	33.0	0.754	2.94
Single-task (4×)	0.903	18.9	32.1	41.0	0.759	9.40

w/o Joint Training	0.896	18.7	31.4	39.6	0.751	3.15
w/o BiP-FPM-Feat	0.902	19.1	32.5	40.7	0.763	3.30
BiP-FPM-Feat (ours)	**0.933**	**23.4**	**39.2**	**46.1**	**0.824**	3.36

Specifically, we compared the full model with the following ablations:

• w/o WTConv: Replacing the wavelet transform convolutional branch with standard 3×3 convolutions, removing explicit frequency-domain modeling.
• w/o FS-FF: Removing the Spatial–Frequency Fusion Module, directly forwarding spatial features to the decoder without domain alignment.
• w/o SE block: Omitting the Squeeze-and-Excitation attention blocks in the decoder, eliminating dynamic channel-wise recalibration.
• w/o DP-IQA: Removing the entropy-aware perceptual supervision module from the decoding path, relying solely on pixel-wise L1 loss.
• Single-task (4×): Training four independent stain-specific networks without a shared encoder, removing inter-task feature sharing.

The full model achieved the best overall performance, including the highest H&E SSIM (0.928), fluorescence intensities (DAPI: 22.8, LAP2: 38.1, panCK: 45.3), and segmentation accuracy (mIoU: 0.812). The removal of either WTConv or FS-FF resulted in the most severe performance drops across all metrics—confirming the importance of explicit frequency modeling and spatial–frequency domain fusion for recovering fine-grained stain structures. Notably, removing DP-IQA led to the lowest DAPI and LAP2 intensities, suggesting that entropy-guided perceptual feedback is essential for preserving high-frequency nuclear signals. Excluding SE blocks degraded channel specificity and stain contrast, while the single-task baseline underperforms due to the lack of multi-stain semantic correlation.

In terms of efficiency, our multi-task design can also offer practical advantages. Despite generating four stain outputs simultaneously, the proposed model achieves an inference time of 3.32 seconds per image—substantially lower than the cumulative time of running four independent single-task networks (2.35 seconds × 4 = 9.4 seconds). This demonstrates that our parallel architecture not only improves biological fidelity but also significantly reduces computational overhead.

In addition to the baseline ablations, we further investigated two key design choices: (1) whether the pipeline is trained end-to-end, and (2) whether BiP-FPM spatial features are integrated into the staining module. The results are summarized in Table 3.

#### Effect of joint training.

When the pipeline was trained in a decoupled manner (*w/o Joint Training*), performance dropped consistently across all metrics (H&E SSIM: 0.896 vs. 0.928; mIoU: 0.751 vs. 0.812). This indicates that end-to-end optimization is essential for aligning the objectives of super-resolution and staining. By allowing gradients from the staining losses to flow into the BiP-FPM encoder, structural features are shaped to better support semantic colorization, while the physics-consistency loss $L_{\text{FPM}}$ prevents overfitting to staining-specific patterns. This synergy results in both higher perceptual fidelity and improved downstream segmentation accuracy.

#### Effect of BiP-FPM feature fusion.

Removing the BiP-FPM spatial features (*w/o BiP-FPM-Feat*) and reverting to an independent ResNet-9 spatial branch led to weaker structural alignment (SSIM: 0.902 vs. 0.933; mIoU: 0.763 vs. 0.824). In contrast, the proposed *BiP-FPM-Feat (ours)* design leverages the multi-scale 9-ResNet encoder features directly from BiP-FPM, ensuring that staining predictions are anchored to physics-constrained reconstructions. This “structural-first” fusion improves nuclear localization in DAPI and membrane delineation in LAP2/panCK, as reflected in higher intensity fidelity scores. Importantly, the computational overhead is negligible (3.36s vs. 3.32s per image), confirming that the feature substitution achieves meaningful gains without sacrificing efficiency.

Overall, the ablation results validate that each component contributes meaningfully to the fidelity, clarity, and efficiency of the generated virtual stains.

## 5. Discussion

While FPM2Stain Net achieves state-of-the-art performance across multiple datasets, it is important to acknowledge model uncertainty and potential failure cases, especially for clinical translation.

### Model Uncertainty.

To quantify prediction reliability, we evaluated two complementary strategies during inference. First, we applied Monte Carlo Dropout (dropout rate p=0.1 ) across 10 stochastic forward passes, producing pixel-level variance maps that highlight low-confidence regions. Second, we introduced Test-Time Augmentation (TTA), including geometric flips and brightness perturbations, and aggregated predictions to derive channel-wise confidence intervals. These approaches allow uncertainty to be visualized as heatmaps or variance overlays on the virtually stained outputs, providing interpretable indicators of confidence that can be integrated into digital pathology workflows.

### Failure Cases and Mitigation.

Despite the robustness of our framework, several scenarios may lead to degraded performance: (i) extremely low signal-to-noise ratio (SNR) acquisitions, (ii) samples with heavy scattering or strong phase artifacts, (iii) tissue types not represented in the training data (e.g., rare cancers, brain or liver tissues), and (iv) severe optical misalignments that induce pupil estimation drift. Under such conditions, the network may generate blurred outputs, hallucinated structures, or reduced stain fidelity. To mitigate these risks, we propose increasing the weight of the physics-consistency loss $L_{\text{FPM}}$ , incorporating Wiener deconvolution priors, and applying domain adaptation or fine-tuning strategies on limited target-domain samples.

### Quality Control.

Finally, to reduce the risk of visually plausible but biologically misleading results, we integrate DP-IQA as a perceptual critic during training. This ensures that predictions are constrained not only by pixel-wise accuracy but also by perceptual fidelity, serving as an internal quality control mechanism. Combined with uncertainty visualization, this enables both quantitative and qualitative assessment of prediction reliability, enhancing trust in clinical applications.

Overall, these additions improve the robustness, interpretability, and safety of FPM2Stain Net, highlighting its potential for reliable integration into real-world digital pathology workflows.

## 6. Conclusions

We presented FPM2Stain Net, a novel end-to-end pipeline that integrates physics-based Fourier Ptychographic Microscopy (BiP-FPM) with a multi-task virtual staining network for high-resolution, label-free digital histopathology. The proposed BiP-FPM module reconstructs amplitude and phase maps from single low-resolution LED images through a self-supervised, physically interpretable learning framework. Building upon this structural foundation, the staining module synthesizes clinically relevant stain modalities—including DAPI, LAP2, panCK, and H&E—using a wavelet-enhanced conditional GAN with spatial–frequency fusion and perceptual supervision.

Extensive experiments on both synthetic and real datasets confirm that FPM2Stain Net achieves state-of-the-art performance in structural reconstruction and digital staining. Ablation analyses further highlight the critical role of physics-informed modeling, spatial–frequency fusion, and semantic constraints in enhancing overall fidelity. Moreover, the synthesized stains not only exhibit strong visual fidelity but also enable accurate downstream analyses such as cell segmentation and biomarker quantification, outperforming traditional microscopy in spatial resolution and diagnostic interpretability.

Experimental results confirm that FPM2Stain Net offers a fast, cost-effective, and scalable alternative to conventional staining workflows, with significant potential for deployment in digital pathology, multiplex imaging, and point-of-care diagnostics. Future work will explore model generalization across tissue types and extend the pipeline to support real-time in situ analysis.

## Supplemental information

Supplement 1 Supplemental Document https://doi.org/10.6084/m9.figshare.31157968

## Data Availability

The data and source code underlying the results presented in this paper are not publicly available at this stage. The source code for FPM2Stain Net, including training scripts and pretrained models, will be integrated into the ImageJ toolbox. Both data and code may be obtained from the authors upon reasonable request for research and academic use.

## Supplemental document

See Supplement 1 for supporting content.
