# Supplementary material for: FPM2Stain Net: physics-guided super-resolution and multi-modal virtual staining for digital histopathology
Source: Biomed Opt Express. 2026 Jan 30;17(2):1074–97. doi: 10.1364/BOE.586327 (PMC12904525; doi:10.1364/BOE.586327)
Supplement: Supplement 1 [file boe-17-2-1074-s001.pdf]

Supplemental Document

Biomedical Optics EXPRESS

FPM2Stain Net: physics-guided super-resolution and multi-modal virtual staining for digital histopathology: supplement

QIJUN YANG,[1,3] LINTAO XIANG,[1] CHANG BIAN,[2] YATING HUANG,[1] HONGPEI ZHENG,[1] AND HUJUN YIN[1,*]

[1] *Department of Electrical and Electronic Engineering, The University of Manchester, Oxford Road, Manchester, M13 9PL, UK*
[2] *Division of Informatics, Imaging and Data Sciences, School of Health Sciences, The University of Manchester, Oxford Road, Manchester, M13 9PL, UK*
[3] *yang.qijun@hotmail.com*
[*] *hujun.yin@manchester.ac.uk*

This supplement published with Optica Publishing Group on 30 January 2026 by The Authors under the terms of the Creative Commons Attribution 4.0 License in the format provided by the authors and unedited. Further distribution of this work must maintain attribution to the author(s) and the published article's title, journal citation, and DOI.

Supplement DOI: https://doi.org/10.6084/m9.figshare.31157968

Parent Article DOI: https://doi.org/10.1364/BOE.586327

FPM2Stain Net: Physics-Guided Single-Shot Super-Resolution and Multi-Modal Virtual Staining for Digital Histopathology

This supplementary document provides detailed implementation, evaluation, and analysis of the proposed **FPM2Stain Net**, an end-to-end framework for single-shot super-resolution and multi-modal virtual staining in digital histopathology. We first present the full architecture of the BiP-FPM module and the multi-task staining network, including wavelet-based spatial-frequency fusion and SE attention mechanisms. Next, we detail the pupil function modeling, data preparation pipeline, and training augmentations. We introduce **DP-IQA**, a novel dual-branch no-reference image quality assessment network tailored for large field-of-view microscopy, along with its architecture, objectives, and validation. Additional ablation visualizations, intensity profile comparisons, and per-cell quantification results are provided to further support the effectiveness and clinical relevance of our approach. Limitations, future directions, and code availability plans are also discussed.

S1. NETWORK ARCHITECTURE DETAILS

BiP-FPM ResNet-U-Net:

Layer	Type	Kernel / Stride	Output Channels
Input	Conv2D	3×3 / 1	64
Encoder 1–3	Conv2D + ReLU + BN	3×3 / 2	128 / 256 / 512
Bottleneck	3 ResBlocks	-	512
Decoder 1–3	Upsample + Conv2D	-	256 / 128 / 64
Output	Conv2D (4 channels)	3×3 / 1	Amplitude, Phase, Pupil, IllumCorr

Table S1. BiP-FPM ResNet-U-Net architecture

Multi-task Staining Network:

- **Spatial branch:** Spatial branch is replaced from the BiP-FPM encoder features 9-ResNet blocks
- **Frequency branch:** WTConv (Wavelet decomposition → 3×3 depthwise conv → IWT)
- **Decoder:** 4 upsampling blocks with SE attention and skip connections
- **Output:** 512×512×4 image (H&E, DAPI, LAP2, panCK)

S2. PSF AND PUPIL FUNCTION MODELING

The pupil function $P(f_x, f_y)$ is modeled with 15-order Zernike polynomials. The coefficients Z_i are trainable parameters regularized by symmetry and smoothness constraints.

This modeling significantly improves robustness under optical aberrations and LED misalignment.

S3. DATA PREPROCESSING AND AUGMENTATION

- **HEMIT:** H&E images cropped to 512×512 patches

- **DeepLIIF:** Multiplex IF channels normalized to [0,1]
- **Augmentation:** Random rotation (0–360°), flipping, brightness jitter (±15%)

Total of ∼150,000 patches were sampled during training.

S4. DIFFERENCE-PERCEPTION IQA NETWORK (DP-IQA)

To improve the robustness and perceptual fidelity of no-reference image quality assessment (NR-IQA), we propose a novel dual-branch framework named **Difference-Perception Image Quality Assessment Network (DP-IQA)**. As illustrated in Figure S1, DP-IQA combines two synergistic learning objectives: (1) absolute quality prediction via a semantic regression path, and (2) relative quality difference modeling via a frequency-aware auxiliary path. The auxiliary branch encodes multi-scale, multi-frequency distortions and generates a difference-aware representation that guides the main regressor toward perceptually relevant degradations.

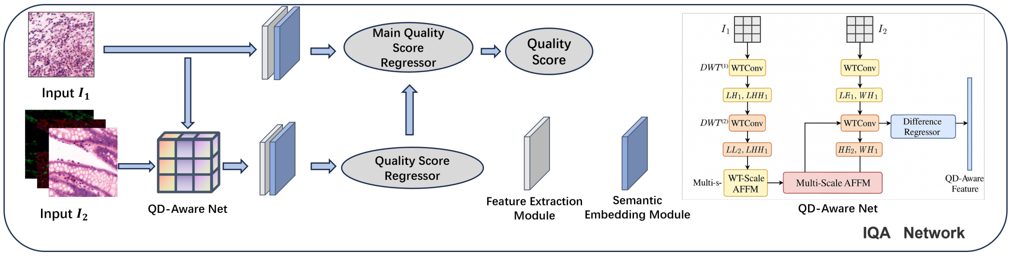

Fig. S1. Structure of Difference-Perception IQA Network (DP-IQA) module

A. Network Overview

DP-IQA consists of two collaborative pathways:

- A **main regression branch** that learns semantic and structural features to directly predict image quality.
- A **difference-aware branch**, which models perceptual differences between image pairs using wavelet-based multi-scale fusion and supplies quality-aware features to refine the main prediction.

The fusion of both pathways enables the model to learn not only how distorted an image is, but also *why* and *where* distortions manifest, closely mimicking human visual assessment.

B. Main Regression Branch

The main branch focuses on extracting image-level features and regressing a quality score through the following components:

- **Feature Extraction Module:** A deep convolutional encoder (e.g., ResNet-50) extracts hierarchical features $F_{\text{main}} \in \mathbb{R}^{C\times H\times W}$ from the input image I.
- **Semantic Embedding Module:** The feature map is globally pooled and optionally enhanced by channel attention to obtain a compact semantic vector $z_{\text{main}} \in \mathbb{R}^d$.
- **Main Quality Score Regressor:** A multilayer perceptron maps z_{main} to a scalar quality prediction:
$$\hat{q}_{\text{main}} = \text{MLP}(z_{\text{main}}) = \text{FC}_3(z_{\text{main}}) \quad \text{(S1)}$$

C. Difference-Aware Auxiliary Branch

The auxiliary branch models perceptual quality differences between image pairs (I_1, I_2) through a structured difference-aware module:

1. **Wavelet Decomposition:** Two-level discrete wavelet transform (DWT) is applied:
$$\text{DWT}^{(1)}(I) \to \{LL_1, LH_1, HL_1, HH_1\},\ \text{DWT}^{(2)}(LL_1) \to \{LL_2, LH_2, HL_2, HH_2\} \quad \text{(S2)}$$

2. **Wavelet Convolution:** WTConv blocks extract frequency-aware features at each level.
3. **Multi-Scale Feature Fusion (MS-AFFM):** Features are adaptively aggregated into a difference-aware representation F_{diff}.
4. **Difference Regressor:** A lightweight regressor predicts perceptual difference:

$$\hat{d} = \text{Reg}_{\text{diff}}(F_{\text{diff}}) \tag{S3}$$

The feature F_{diff} is also injected into the main regressor to enhance its quality prediction.

D. Multi-Scale Asymmetric Feature Fusion Module (MS-AFFM)

To handle the complexity of multi-scale frequency information, we introduce MS-AFFM for adaptive fusion:

- **Upsampling:** All features are spatially aligned:

$$\tilde{F}^{(s)} = \text{Upsample}(F^{(s)}) \tag{S4}$$

- **Asymmetric Attention:**

$$\alpha_s = \sigma(W_2 \cdot \delta(W_1 \cdot \text{GAP}(\tilde{F}^{(s)}))) \tag{S5}$$

- **Weighted Fusion:**

$$F_{\text{fused}} = \sum_{s=1}^{S} \alpha_s \odot \tilde{F}^{(s)} \tag{S6}$$

- **Residual Enhancement (optional):**

$$F_{\text{final}} = \text{Conv}(F_{\text{fused}}) + F_{\text{fused}} \tag{S7}$$

E. Training Objective

DP-IQA is trained end-to-end using a joint loss:

$$\mathcal{L}_{\text{IQA}} = \mathcal{L}_{\text{main}} + \lambda_1 \mathcal{L}_{\text{aux}} + \lambda_2 \mathcal{L}_{\text{diff}} \tag{S8}$$

where:

$$\mathcal{L}_{\text{main}} = \|\hat{q}_{\text{main}} - q\|_1 \tag{S9}$$

$$\mathcal{L}_{\text{aux}} = \|\hat{q}_{\text{aux}} - q\|_1 \tag{S10}$$

$$\mathcal{L}_{\text{diff}} = \|\hat{d} - |q_1 - q_2|\|_1 \tag{S11}$$

λ_1 and λ_2 are loss weights (for example, 0.5 and 1.0).

F. DP-IQA Neural Network Validation

To verify the effectiveness of IEDP-IQA in large field-of-view and complex scenes, we perform experiments on two representative IQA datasets, LIVE [1] and KADID [?], and compare it with seven other state-of-the-art (SOTA) no-reference methods.

Table S2. Performance comparison on LIVE and KADID datasets.

	LIVE		KADID	
	PLCC	SRCC	PLCC	SRCC
DIIVINE [2]	0.902	0.887	0.433	0.415
BRISQUE [3]	0.933	0.921	0.557	0.519
IQDLNet [4]	0.977	0.970	0.866	0.857
MetaIQA [5]	0.957	0.961	0.773	0.759
HyperIQA [6]	0.963	0.961	0.825	0.852
VCRNet [7]	**0.979**	0.973	0.856	0.853
DP-IQA	0.964	**0.977**	**0.896**	**0.893**

From Table S2, we can see that the IEDP-IQA model has a significant improvement over the non-perceptual IE-IQA network when facing complex and large-scale targets.

DP-IQA effectively combines absolute and relative quality supervision, integrates wavelet-based multi-scale frequency modeling, and incorporates difference-aware representations to improve the perceptual alignment of quality predictions.

S5. ADDITIONAL ABLATION VISUALIZATION

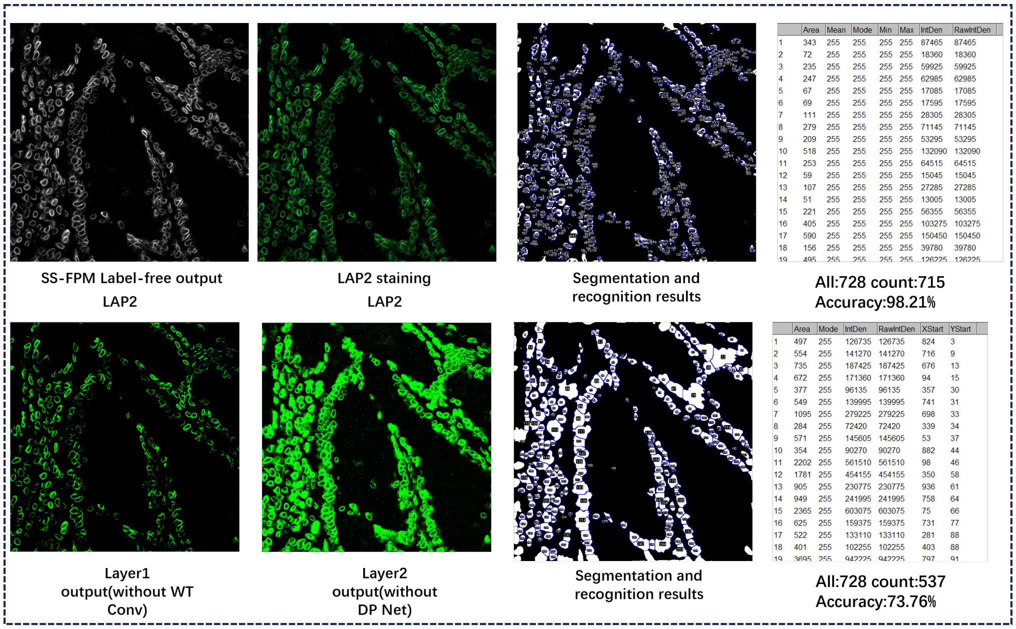

Fig. S2. Top: Original grayscale and virtually stained LAP2 images, with Cellpose-based segmentation and intensity measurements. Bottom: Predicted stain, segmentation overlay, and per-cell quantitative outputs from ImageJ. These results demonstrate accurate nuclear envelope localization and high-fidelity cell-level quantification.

S5. ADDITIONAL STAINING ANALYSES

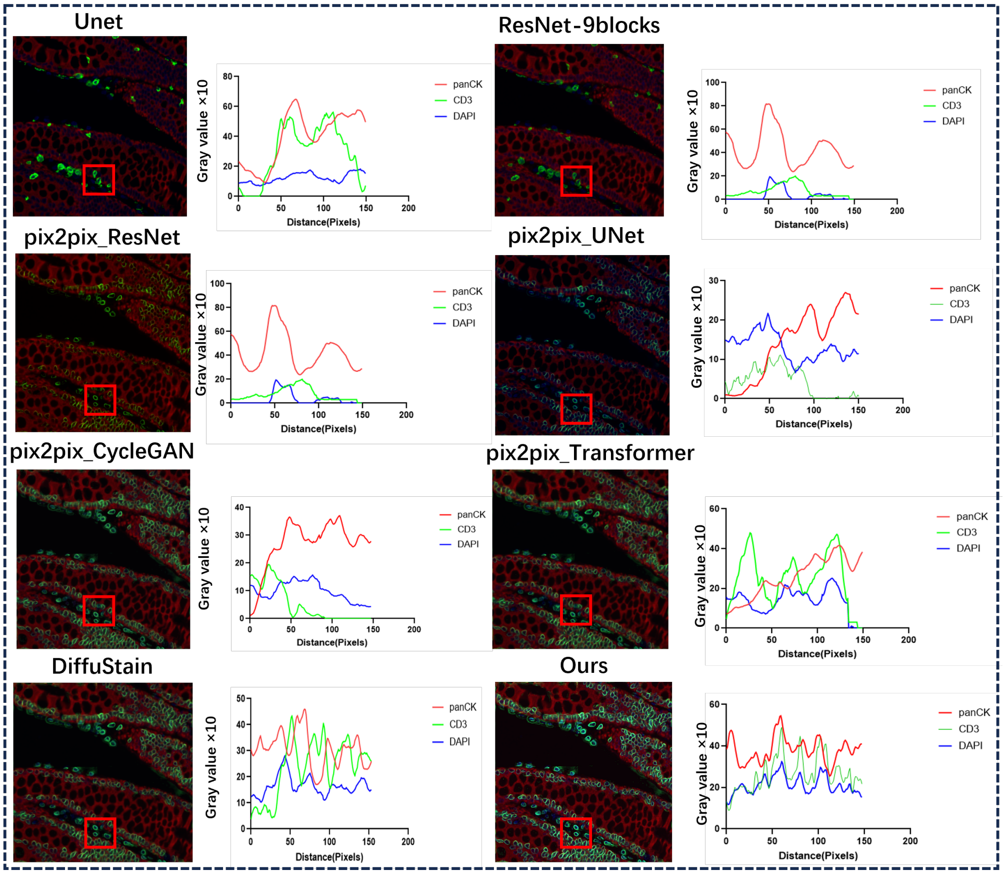

Fig. S3. Line intensity profiles across panCK, CD3, and DAPI channels. Each row shows spatial line plots sampled from virtual (left) and real (right) multiplex stains, with red boxes indicating the profile region. The intensity curves demonstrate consistent structural localization across stains, validating the fidelity of FPM2Stain Net predictions.

S6. LIMITATIONS AND FUTURE WORK

- **Generalizability:** Model performance on rare tissue types (e.g., brain, liver) is yet to be validated.
- **Scalability:** Although 4 stain types are supported, further extension may require large, balanced datasets.
- **Inference efficiency:** The current 3.32 s/image latency may be suboptimal for real-time workflows.

Future directions include semi-supervised domain adaptation, multi-center validation, and integration with interactive histology viewers.

S7. CODE AVAILABILITY

The source code for **FPM2Stain Net**, including training scripts and pretrained models, will be made publicly available upon paper acceptance. We will also integrate the virtual staining module as a plugin in the **ImingJ** toolbox to support wider use in open-source digital pathology workflows.

REFERENCES

1. H. R. Sheikh, M. F. Sabir, and A. C. Bovik, "A statistical evaluation of recent full reference image quality assessment algorithms," IEEE Transactions on image processing **15**, 3440–3451 (2006).
2. M. A. Saad, A. C. Bovik, and C. Charrier, "Blind image quality assessment: A natural scene statistics approach in the dct domain," IEEE transactions on Image Process. **21**, 3339–3352 (2012).
3. L. Zhang, L. Zhang, and A. C. Bovik, "A feature-enriched completely blind image quality evaluator," IEEE Transactions on Image Process. **24**, 2579–2591 (2015).
4. J. Xie, Y. Luo, J. Ling, and G. Yue, "No reference image quality assessment via quality difference learning," in *2023 IEEE International Conference on Multimedia and Expo (ICME),* (IEEE, 2023), pp. 1301–1306.
5. H. Zhu, L. Li, J. Wu, *et al.*, "Metaiqa: Deep meta-learning for no-reference image quality assessment," in *Proceedings of the IEEE/CVF conference on computer vision and pattern recognition,* (2020), pp. 14143–14152.
6. S. Su, Q. Yan, Y. Zhu, *et al.*, "Blindly assess image quality in the wild guided by a self-adaptive hyper network," in *Proceedings of the IEEE/CVF conference on computer vision and pattern recognition,* (2020), pp. 3667–3676.
7. Z. Pan, F. Yuan, J. Lei, *et al.*, "Vcrnet: Visual compensation restoration network for no-reference image quality assessment," IEEE Transactions on Image Process. **31**, 1613–1627 (2022).
